# Numbers Driving Space: Experience With Numerical Sequences Modulates Spatial Behavior in Newborn Chicks (*Gallus gallus*)

**DOI:** 10.1111/nyas.70325

**Published:** 2026-07-14

**Authors:** Arianna Felisatti, Matteo Macchinizzi, Lucia Ronconi, Rosa Rugani

**Affiliations:** ^1^ Department of General Psychology University of Padua Padua Italy; ^2^ Computer and Statistical Services, Multifunctional Pole of Psychology University of Padua Padua Italy

**Keywords:** comparative psychology, domestic chicks (*Gallus gallus*), left‐side bias, mental number line, numerical cognition, spatial−numerical association, visuospatial functions

## Abstract

The preferential engagement of the right hemisphere in vertebrates biases visuospatial attention to the left. This attentional bias has been proposed as the basis for the organization of numbers along a left‐to‐right mental number line, with small numbers associated with the left and large ones with the right. In humans, this predisposition is modulated by culture, education, and even short exposure to spatial positions of numbers; yet data on animals are lacking. Here, in day‐old chicks, we explore whether experience with increasing and decreasing numerical sequences influences the left‐to‐right visual exploration of numerosities. We conducted two experiments with day‐old chicks (*n* = 70), training them to circumnavigate a sagittal sequence of three number sequences. In Experiment 1, chicks saw stimuli with either 2‐5‐8 (increasing‐group) or 8‐5‐2 (decreasing‐group) elements and then evaluated for searching behavior from left to right; in Experiment 2, we used larger numerosities, 8‐20‐32 or 32‐20‐8, and controlled for non‐numerical cues. At test, chicks faced the stimuli arranged horizontally. We found that the increasing‐group consistently approached the number stimuli from the left, regardless of the number arrangement. In contrast, the decreasing‐group showed no directional preference. These results demonstrate that early exposure to numerical sequences can modulate the spontaneous left‐to‐right bias in newborn chicks, shedding light on the flexibility of cognitive biases in visuospatial and numerical cognition early in life.

## Graphical abstract

Chicks learned to circumnavigate a sagittal series of stimuli displaying increasing or decreasing numerosities. When tested with the series arranged horizontally, chicks trained on increasing numerosities maintained the spontaneous left‐to‐right approaching behavior, while those trained on decreasing numerosities lost this bias. The effect generalized across numerical ranges and was independent of continuous variables. This study reveals that numerical experiences modulate spontaneous left‐to‐right behavior even in newborn animals.

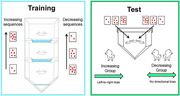

## 1 Introduction

When asked to bisect horizontal lines, we typically misallocate the central point to the left [1]. This left‐side bias emerges early in human development. Infants as young as 4 and 5 months old exhibit a spontaneous leftward gaze bias when viewing horizontal lines [2]. This suggests that asymmetries in visuospatial attention are present early in life, potentially indicating a pre‐existing or rapidly developing bias in spatial processing. The left‐side bias extends to avian species. In a cancellation task involving the retrieval of food grains within a designated area, chicks (*Gallus gallus*) and pigeons (*Columba livia*) demonstrated a consistent left‐side bias [3]. Further research corroborated these findings by adapting the classic human line bisection task [1, 2] to chicks [4]. After being trained to peck at the center of a line, chicks reported a consistent tendency to start searching from the left edge at test. This attentional bias was more pronounced in chicks with stronger brain lateralization and maturation of the right hemisphere [5].

Importantly, the left‐side bias also emerges when processing numerical information [5, 6, 7, 8, 9], as evidenced by comparative [5] and developmental studies [10, 11, 12]. In a proto‐counting direction task, day‐old chicks learnt to identify a target element based on its ordinal position (e.g., the fourth) within a series of 10 identical elements. At test, the sequence was rotated by 90° and oriented horizontally from left to right. Both the fourth element from the left and the fourth from the right were correct options; nonetheless, chicks preferentially selected the fourth left element [5, 13]. This left‐to‐right searching behavior only appears in strongly brain‐lateralized chicks [6], indicating the critical role of hemispheric lateralization. This form of spatial−numerical association has been interpreted as indicative of a spatial representation of numerical information (ordinal and cardinal) along a horizontal mental number line (MNL), typically oriented from left to right [14, 15, 16]. This orientation aligns with the left‐side attentional bias observed in visuospatial tasks, suggesting a fundamental connection between spatial attention and numerical cognition. The left‐side bias in spatial−numerical mapping also emerged in Clark's nutcrackers (*Nucifraga columbiana*) [13] and rhesus monkeys (*Macaca mulatta*) [17]. Notably, in monkeys, spatial−numerical association is modulated by the numerosity of the array. In a touch‐screen task requiring them to remember the location of visual targets, monkeys remembered better left targets when the array is composed of two items, and right targets when the array is composed of six or 10 items [18]. Indeed, besides ordinal information, numerosities are also mapped from left to right in a manner that resembles the human MNL. Various animals, including chicks, reveal a preference for associating smaller numerosities with the left and larger numerosities with the right [16, 19, 20, 21, 22].

Also, human infants report a spontaneous preference for sequences increasing from left‐to‐right [10]. Notably, this spontaneous preference is modulated by early experiences. Infants habituated to temporal sequences displaying increasing or decreasing numerosities from left to right on a screen showed a significant preference for the increasing sequence. In contrast, infants habituated to similar sequences but displayed from right to left did not show preference for either increasing or decreasing sequences.

Notably, this left‐to‐right mapping of numerical information displays developmental variation, as revealed by the performance of 1‐ to 5‐year‐old children in a proto‐counting direction task. Children were exposed to a video where an object disappeared behind one of several vertically aligned items [12]. Then, the item arrangement was rotated by 90°, resulting in a horizontal arrangement. Analyses of eye movements revealed that left‐to‐right search behavior was already present in infants as young as 1 year, but the behavior fluctuated with age. This indicates a dynamic developmental trajectory of spatial−numerical association, potentially reflecting an interplay between precociously available biases and educational influences (i.e., explicit acquisition of mathematical and symbolic knowledge) [16, 23].

Also, exposure to cultural routines (i.e., implicit, overlearned scanning habits that shape spatial attention even prior to or independent of formal mathematical education) plays a significant role [8, 24]. This has been documented using dot counting tasks, where participants are required to count dots in a horizontal array by pointing to or clicking on each of them [24, 25]. Even prior to formal schooling, exposure to culture‐specific scanning routines can determine counting direction [24]. Illiterate adults from villages in northern Ethiopia, for example, show no directional preference for counting dots; children from left‐to‐right reading cultures consistently count from left to right; and children from right‐to‐left reading cultures count starting from the right [24]. Notably, as children acquire the ability to read both text and numbers in school, counting preferences become stronger in cultures where text and number reading directions are consistent, compared to to cultures where text and number reading directions diverge. This is exemplified by Israeli culture, where text is read from right to left but numbers are read from left to right; mixed reading habits result in inconsistent counting direction preferences [8, 24] (for a review, see Ref [26]). These findings have been corroborated and extended by the administration of card sorting tasks requiring people to order a set of numbers instead of single dots. Educated adults consistently map numbers from left to right [27]. In contrast, preschoolers and Himba adults (a native population with limited mathematical knowledge and no formal schooling) do not show a consistent group preference. Also, when opening the selection of both direction and axis to participants, Tsimanè individuals (an indigenous tribe living in Bolivia) select more often a sagittal mapping, regardless of the level of education, while people from industrialized societies prefer far‐left to near‐right diagonal mapping and horizontal mapping [28].

Not only extensive experience with culture‐specific learning‐related practices, but even brief exposure to a specific arrangement of numbers can rapidly change how they are mentally associated with space. For example, after reading a text reporting a disposition of numbers incongruent with the reading direction habits, when asked to classify digits as odd or even, English participants reported reduced spatial−numerical association, while Hebrew participants reported a reversed mapping [29].

This brief overview suggests that (1) individuals have a predisposition to count increasing numerosities from left to right; and (2) cultural practices, formal instruction, and brief exposure can modulate this directional preference. Notably, to date, the influence of long‐ and short‐term experience on spatial and numerical processing has only been documented in humans (both preverbal and verbal) and never in nonverbal populations.

Here, in two experiments, we tested day‐old domestic chicks to determine whether early exposure to increasing or decreasing numerical sequences modulates the spontaneous searching behavior from left to right. We selected the domestic chick as an animal model because (1) chicks exhibit a left‐side bias in visuospatial tasks [3, 4, 30] and a left‐to‐right association of numerosity and ordinal information [5, 6, 20, 31]; (2) chicks lack a corpus callosum homolog, resulting in pronounced brain asymmetries; and (3) chicks possess advanced visual and motor abilities immediately post‐hatching, allowing for nature versus nurture investigations [32].

Exploring whether and how early experience can modulate spatial and numerical processing in day‐old animals may provide the first evidence that experience‐dependent modulation of spatial−numerical association emerges in the absence of language, cultural transmission, and extended postnatal development. Filling these gaps would offer critical insights into the evolutionary roots and early flexibility of cognitive biases in visuospatial and numerical cognition.

## 2 Experiment 1

In Experiment 1, two groups of chicks learned to circumnavigate a sagittal series of three sequential (near‐far) panels displaying 2, 5, or 8 elements in either increasing (2‐5‐8; increasing‐group) or decreasing (8‐5‐2; decreasing‐group) order. At test, they faced the same numerosities arranged horizontally from left to right, either in an MNL‐congruent (2‐5‐8) or MNL‐incongruent (8‐5‐2) arrangement.

All experimental procedures were approved and conducted in strict adherence to the guidelines provided by the Committee for Animal Welfare of the University of Padua, the Ethical Committee of the University of Padua for Animal Experimentation, and the Ministry of Health of the Italian Republic (Prot. N.269/2025‐PR, 10/04/2025). This comprehensive compliance addressed both national and European directives concerning animal research.

### 2.1 Materials and Methods

#### 2.1.1 Subjects and Rearing Conditions

We tested 40 male chicks (*G. gallus*) of the Aviagen ROSS 308 strain, obtained from a commercial hatchery (Società Agricola La Pellegrina Spa, San Pietro in Gù, Padova, Italy). An a‐priori power analysis was conducted using G*Power (version 3.1.9.4) to determine the appropriate sample size for the study. The following parameters were established: Statistical test = repeated measures analysis of variance (ANOVA), within–between interaction; power  =  0.85; α  =  0.05 (medium effect size); f  =  0.25; number of groups = 2 (increasing‐group, decreasing‐group); number of measurements = 2 (MNL‐congruent test, MNL‐incongruent test). We determined that a sample size of at least 38 subjects was required. The analyses conducted employed generalized linear mixed models (GLMMs), which better account for individual differences and the complex structure of the data. Given that parameters selected for the ANOVA offer a general estimation and that GLMMs frequently provide enhanced flexibility and power in detecting effects, we considered the power analysis to be a conservative estimation.

Male chicks were exclusively used due to their superior responsiveness to food reinforcement compared to females, who exhibit greater sensitivity to social stimuli [33, 34].

The chicks were hatched in the Comparative Cognition Laboratory of the Department of General Psychology at the University of Padua. Upon delivery to the lab, eggs were placed in a FIEM incubator MG 70/100 (45 × 58 × 43 cm) at a controlled temperature of 36–38°C and 60% humidity. On the 18th day of incubation, eggs were moved to a VICTORIA hatching machine (60 × 32 × 40 cm) at controlled temperature (36–38°C) and humidity (70%) and exposed to continuous light using an LED 4.8W lightbulb until the 21st day of incubation (hatching day). Light stimulation during the last 3 days of incubation is known to enhance lateralization in chickens, with effects observable in cognitive and behavioral asymmetries after hatching [6, 35, 36].

A few hours post‐hatching, chicks were feather‐sexed and housed in pairs in standard metal cages (28 × 32 × 40 cm) with the floor lined with a paper towel. The rearing room was maintained at a temperature of 28–31°C and the humidity at 50% to ensure an appropriate environment. Illumination was provided by fluorescent lamps (36W) positioned 45 cm above the cages. An automated lighting system controlled the light/dark cycle, with lights on from 7 a.m. to 7 p.m., and alternating light/dark cycles every 2−3 h during the night. Water and high‐grain food (chick crumbles) were available ad libitum, in a water and food jar (5 cm in diameter, 5 cm high) positioned symmetrically within the cage. During the first 2 days post‐hatching, chicks were fed twice a day with three mealworms (*Tenebrio molitor*) to familiarize them with this food, later used during reinforcement during the experimental procedure (for a similar procedure, see Refs [6, 19, 20]). Chicks that were reluctant or refused to eat the worms during the first days were excluded from the experiment. The rearing conditions were kept until the third day after hatching, when the experimental protocol began. On the fifth day post‐hatching, the experiment protocol was completed, after which the chicks were donated to local farmers.

#### 2.1.2 Apparatus

**FIGURE 1:**
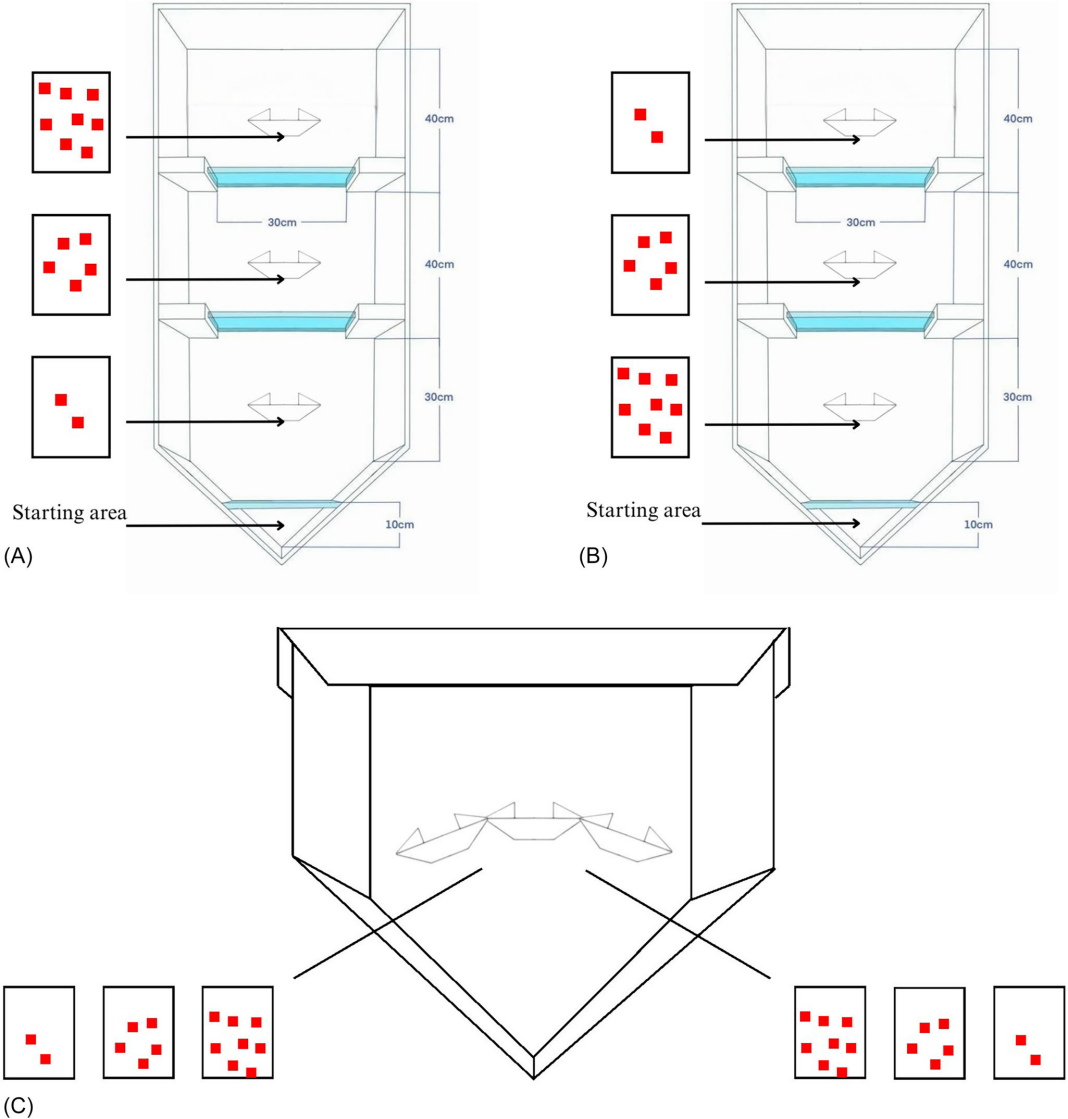
Schematic representation of the apparatus used during: (A) training with an increasing sequence (2‐5‐8); (B) training with a decreasing sequence (8‐5‐2); and (C) testing with stimuli arranged from left to right in an MNL‐congruent (2‐5‐8) or MNL‐incongruent (8‐5‐2) placement.

The experimental setup was in a room near the rearing room. Ambient conditions were maintained at 28°C and 50% humidity throughout experimental procedures. The apparatus consisted of three connected sections separated by transparent, liftable plexiglass barriers (30 × 40 cm; Figure 1A,B). The walls of the apparatus were made of opaque green plastic, with the floor covered with wood chips.

The first section included a triangular area (10 × 30 × 40 cm), referred to as the “starting area”, connected to an adjacent rectangular‐shaped area (30 × 50 × 40 cm) by the first liftable barrier. A white plastic panel displaying a specific stimulus (see Section 2.1.3) was placed 15 cm from the first barrier and 25 cm from the side walls. The second and third sections were rectangular‐shaped (40 × 50 × 40 cm). Each contained a white plastic panel, positioned 15 cm from the barrier and 25 cm from the side walls.

Barriers moved vertically along guides built into the side walls and were operated manually via a pulley system aligned with the sagittal axis. Nylon cables were attached to the top edge of each barrier, passing through metal pulleys (2 cm in diameter) mounted on an external frame. Once lifted, each barrier allowed the animal to move freely from one section to the next. The system was operated from outside the arena to prevent the animals from perceiving any visual cues. During shaping and testing (see Section 2.1.4), the first section was divided from the rest of the apparatus by a green plastic panel (50 × 40 cm).

At test, the three white plastic panels displaying numerical stimuli were placed as follows: A central panel was positioned 15 cm from the barrier and 25 cm from the side walls; two side panels were placed adjacent to the central panel, one on the right and one on the left (Figure 1C); each side panel was rotated 30° relative to its adjacent edge, resulting in an equal distance between the barrier and every panel.

#### 2.1.3 Stimuli

**FIGURE 2:**
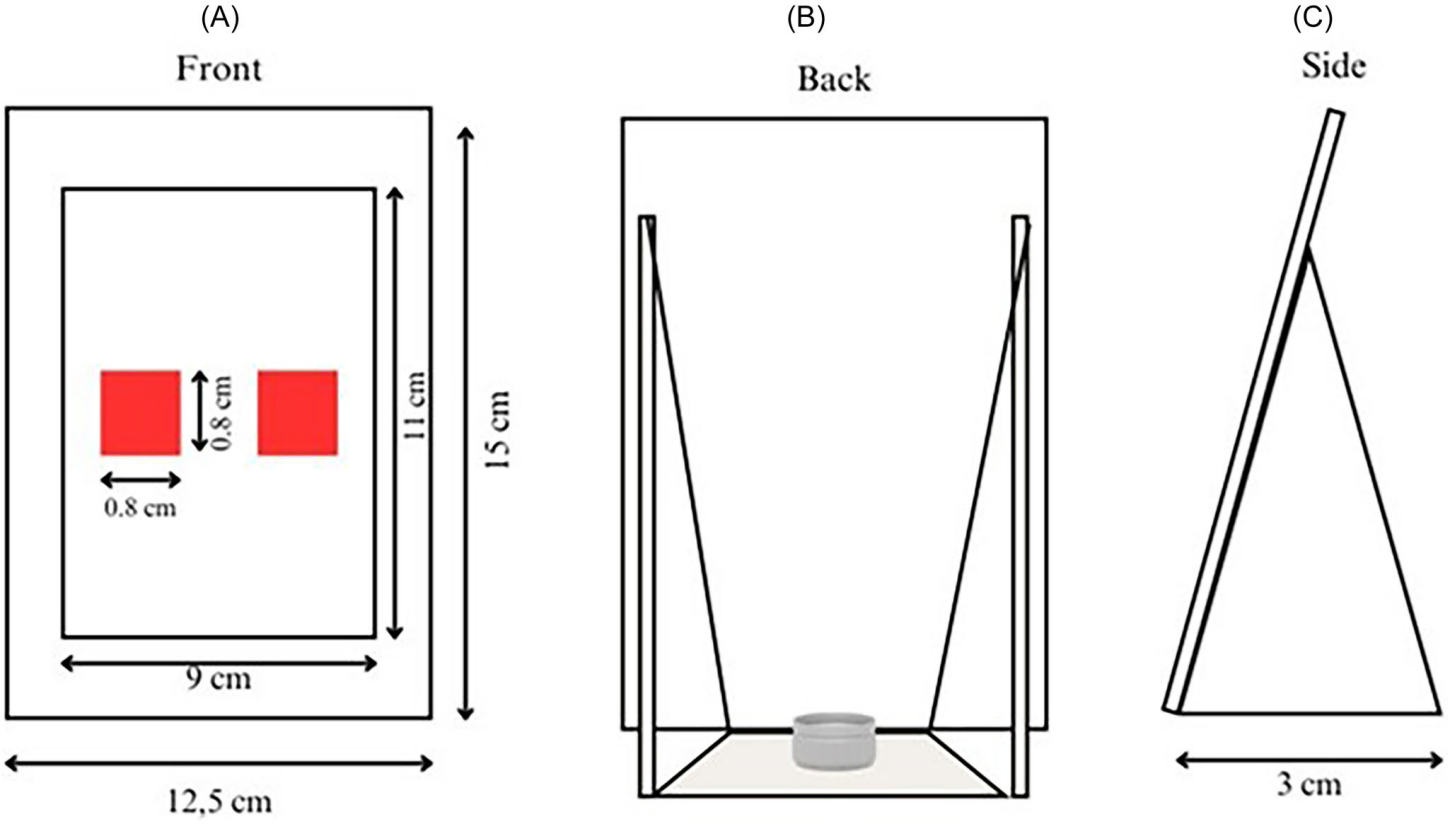
Schematic representation of a panel from the front (A), back (B), and side (C).

The stimuli consisted of static 2D images (11 × 9 cm) attached to the front of a white plastic panel (15 × 12.5 cm; Figure 2A). On the back of each panel, a bottle cap was fixed at the base to hold the food reward (Figure 2B). A 3 cm bent side was added on both sides of the panel to prevent the chicks from seeing the bottle cap before they had circumnavigated it (Figure 2C).

Each image depicted a different number of red squares (0.8 × 0.8 cm): 2, 5, or 8. For stimulus of 2, the minimum and maximum distances between the center of two squares were 3 and 5 cm, respectively; for stimulus of 5, they were 3 and 8.7 cm; and for stimulus of 8, they were 2.5 and 10 cm. The stimuli were placed 15 cm from the transparent barrier. At this distance, each 0.8 cm square subtends a visual angle of 3.05°. This is well within the visual acuity limits of domestic chicks, which reach approximately 7.0 cycles per degree within the first week post‐hatching [37, 38]. Even at the maximum distance from the starting area vertex (25 cm), the stimuli subtended 1.83°, remaining significantly above the detection threshold.

To prevent the chicks from learning the stimuli based on spatial configuration, each training and test period entailed a different spatial arrangement of the squares for each numerosity. Specifically, a total of seven different spatial configurations of each numerosity were used, one for each training and test phase. The different copies of each stimulus were randomly assigned to the five training phases and two test phases (see Section 2.1.4).

#### 2.1.4 Procedure

**FIGURE 3:**
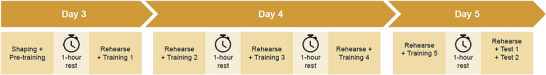
Weekly schedule of the experiment.

The experimental procedure started on the third day after hatching and comprised a shaping phase, a pretraining phase, five training phases, and two tests (Figure 3). Two hours before the beginning of each daily procedure, chicks were food‐deprived to increase their motivation. During training, all trials but two were reinforced with food to maintain the chicks’ focus on the task and prevent the extinction of responses when encountering unrewarded trials at test [6]. During testing, no trial was reinforced. Following the last daily session, chicks were rehoused with water and food ad libitum.

##### 2.1.4.1 Shaping

Shaping was conducted in the first section of the apparatus (see Figure 1) using a single white panel (without stimuli attached) placed in the center of the section, 30 cm from the vertex. For a couple of minutes, the chick was free to explore the novel environment and get acquainted. At the beginning of each shaping trial, a chick was placed in the starting area, confined by the transparent barrier, for a period of 5 s. During this time, a mealworm was placed progressively closer to the white panel over five trials (approximately at 5, 10, and 15 cm in front of the panel, then beside the panel, and finally behind the panel). Initially, a metal stick (35 cm long) was used to guide the chick toward the correct location. As trials progressed, the use of the stick gradually reduced. The shaping phase continued until the chick successfully found the mealworm behind the panel in three consecutive trials without the help of the stick [39]. During the interval between successive trials, chicks were placed in an opaque white box (20 × 40 × 30 cm) for a couple of minutes. After the end of the shaping, the chicks rested for 1 h.

In this as well as in the subsequent experimental phases, whenever a chick showed signs of fatigue, excessive distress (emitting distress calls), or lack of motivation, the session was terminated. Fatigue was operationalized as three consecutive trials with the chick being unresponsive for 1 min. In these cases, the chick would be brought back to the rearing room to rest. After at least 1 h, the chick would be reintroduced to the interrupted experimental phase. The shaping phase lasted between 5 and 10 min.

##### 2.1.4.2 Pretraining

In this phase, the entire apparatus was used and the chick was trained to navigate around the three panels, one located in each of the three sections. Each panel displayed the same stimulus on its front and back faces. The three panels were arranged in increasing (2‐5‐8, Figure 1A) or decreasing (8‐5‐2, Figure 1B) order, depending on the experimental group. Each chick was pseudorandomly assigned to either the increasing‐group or decreasing‐group. The assignment order was initiated arbitrarily, with the first chick assigned to the increasing‐group and then alternated such that each subsequent chick was allocated to the decreasing‐group. This contingency remained fixed for each individual throughout all experimental phases. Twenty chicks underwent pretraining and training with the increasing sequence, and 20 chicks underwent the same procedure with the decreasing sequence.

In the first trial, after circumnavigating the first panel, the chick was attracted with a visible mealworm (that was also indicated using a stick) toward the second panel. Once it reached the transparent partition before the second panel, the chick waited for 5 s behind it. Then, the partition was lifted to allow the chick to approach the second panel. Once it circumnavigated this second panel, the chick was attracted with a visible mealworm toward the third panel. Once it reached the third transparent partition, the chick waited for 5 s behind it. Then, the partition was lifted to allow the chick to approach the third panel. From trial 2, a mealworm was hidden behind all panels before the beginning of each trial. Also, in the pretraining phase, the stick was initially used to guide the chick toward the correct location. A trial was considered completed when the chick circumnavigated all three panels and found the hidden mealworms without the help of the stick. Pretraining ended once the chick successfully completed three consecutive trials. This achievement was considered the learning criterion. The pretraining typically lasted 15−20 min. After 1‐h rest, the training phase began (see Figure 3).

##### 2.1.4.3 Training

Each training phase was preceded by a short rehearsal phase (duration around 5 min) designed to allow the chick to remember the task after the rest. This phase required the chick to successfully circumnavigate the three panels, one by one, three consecutive times. Each training trial started with the chick confined in the starting area behind the transparent barrier for 5 s, to ensure that they processed the stimuli [40]. From the starting area, the chicks could only see the first panel.

Then, the partition was removed, and the chick could enter the first section. In each section, the chick had to circumnavigate the panel to eat a mealworm. Then, the chick was confined for another 5 s behind the second transparent partition before accessing the next section. Once the chick entered the second section, the first transparent partition was covered with a green plastic panel to prevent the chick from viewing the previous panel and attempting to return to it. The same procedure was repeated to access the final section. The trial ended once the chick had circumnavigated the last panel. A trial in which the chick did not eat any mealworm was considered incorrect and was repeated. Each training session comprised seven correct training trials. After the completion of the training session, chicks could rest for 1 h with access to water but no food.

In total, chicks performed five training sessions: Training 1 on the third day after hatching, trainings 2, 3, and 4 on the fourth day after hatching, and training 5 on the fifth day after hatching (see Figure 3). In all training sessions, we used the same numerosities; however, between one training session and the next, we used numerical stimuli that varied in the spatial configurations of the elements (see Section 2.1.3). Each training lasted about 15−20 min.

##### 2.1.4.4 Test

One hour after training 5, each chick underwent two tests. Both tests took place in the first section of the apparatus and involved three panels similar to those used during training (Figure 1C). At test, the three panels were placed horizontally in front of the chick. The two side panels were rotated by 30° relative to the central panel to ensure that the distance from the starting area to each panel was exactly 15 cm for all stimuli. The interpanel distance was 0.5 cm, allowing the chick to approach the three numerical stimuli either from the left or the right side and never from the middle. A numerical stimulus was attached to each panel, with numerosities organized from left to right in either an MNL‐congruent (2‐5‐8; MNL‐congruent test) or MNL‐incongruent (8‐5‐2; MNL‐incongruent test) arrangement.

Test order was pseudorandomly counterbalanced across subjects. In both groups (increasing‐group: *n* = 20; decreasing‐group: *n* = 20), half of the chicks completed the MNL‐congruent test before the MNL‐incongruent test, and vice versa.

At the beginning of each trial, the chick was placed behind the transparent partition for 5 s, to let it observe the stimuli. Afterward, it was released and given a maximum of 1 min to circumnavigate the panels either from the right or left side. A choice was defined as the chick positioning its head and three‐quarters of its body behind a panel (“valid trial”, see Refs [19, 20, 41]). If no choice was made within 1 min, the trial was considered null, and then repeated. Each trial could be repeated a maximum of three consecutive times. After three null trials, a chick would be placed back in the rearing room for 30 min to rest before trying the test again. Each test consisted of three valid trials. Each test phase had a duration of 5−10 min.

On each test trial, we recorded the first panel circumnavigated by the chicks. The two tests were video recorded to observe the chicks from a monitor connected to the video camera (Sony HDR‐CX405, 25 FPS), without interfering with the chicks’ choices. Videos were scored offline by an experimenter blind to the experimental hypotheses.

#### 2.1.5 Design and Hypotheses

Three independent variables were examined: *Group*, a between‐subjects variable with two levels (Increasing‐group, Decreasing‐group); *Test*, a within‐subjects variable with two levels (MNL‐congruent test, MNL‐incongruent test); and *Test order*, a between‐subjects variable with two levels (MNL‐congruent test first, MNL‐incongruent test first).

If exposure to increasing or decreasing numerical sequences influenced the spatial behavior of day‐old chicks, three hypotheses can be generated:

H1 Numerical training affects behavior from the orientation of the MNL. This is the only hypothesis that predicts a significant test effect (a difference between congruent‐MNL and incongruent‐MNL tests):
  ‐ The increasing‐group would circumnavigate the panels from the left, especially in the MNL‐congruent test, due to congruency between increasing numerical training (small‐first to large‐last) and the MNL;
  ‐ The decreasing‐group would show a preference for circumnavigating panels from the right, particularly in the MNL‐incongruent test, due to incongruency between the decreasing numerical training (large‐first to small‐last) and the MNL.
H2 Numerical training affects spontaneous left‐side visuospatial bias. This hypothesis predicts that the right‐to‐left activation from decreasing training conflicts with the spontaneous left‐side bias, leading to a reduced or null rather than reversal spatial preference. Unlike hypothesis 1, it predicts that behavior will be independent of the stimuli arrangement at test:
  ‐ The increasing‐group would maintain a left‐to‐right preference, as the training sequence (small‐to‐large) aligns with the spontaneous left‐side bias;
  ‐ The decreasing‐group would show a reduced or no lateral bias, due to conflict between the right‐to‐left numerical activation induced by training and the spontaneous left‐side bias.
H3 Numerical training fully replaces the spontaneous left‐side bias. Like H2, this hypothesis predicts that behavior will be independent of test arrangement, unlike H2, it postulates a full behavioral reversal rather than a simple reduction or cancellation:
  ‐ The increasing‐group would preferentially circumnavigate panels from the left, regardless of the test (MNL‐congruent and MNL‐incongruent);
  ‐ The decreasing‐group would preferentially circumnavigate panels from the right, regardless of the test.

#### 2.1.6 Analyses

The analyses for Experiment 1 were conducted on data from 40 chicks (increasing‐group: *n* = 20; decreasing‐group: *n* = 20), with a total of 240 data points (six trials per subject).

The dependent variables were: (1) *Circumnavigation Side*, a binary trial‐level measure coded as “1” for left and “0” for right, which allows for the analysis of individual choices within GLMMs while accounting for subject‐specific variability; and (2) *Left Preference Index*, computed by the following formula: (number of circumnavigations of the panels from left / number of total trials) × 100. This index provides a summary score of overall performance and was used for nonparametric tests and to evaluate group‐level preferences against the 50% chance level.

We first ran GLMMs on Circumnavigation Side with a binomial family and logit link function. We conducted both frequentist and Bayesian GLMMs. The models included Group (increasing‐group vs. decreasing‐group), Test (MNL‐congruent test vs. MNL‐incongruent test), and Test order (MNL‐congruent test first vs. MNL‐incongruent test first) as fixed factors, and Subject as a random factor.

To make specific comparisons, primary analyses were complemented by nonparametric tests on Left Preference Index. Specifically, to analyze differences from chance level (50%), we performed the Wilcoxon signed‐rank test for one‐sample. For post‐hoc comparisons, we conducted the Mann−Whitney test for two independent samples and the Wilcoxon signed‐rank test for two paired samples.

To quantify the evidence for our hypotheses, we also complemented the frequentist post‐hoc *t*‐tests with Bayesian analyses: Bayesian Wilcoxon signed‐rank tests for differences from chance level and within‐sample comparisons, and Bayesian Mann−Whitney tests for between‐sample comparisons. Bayes factors (BF) were interpreted using the classification by Lee and Wagenmakers [42], where values are categorized as follows: 1–3 (anecdotal), 3–10 (moderate), 10–30 (strong), 30–100 (very strong), and >100 (extreme) evidence for the alternative hypothesis (*H_1_*). Conversely, BF_10_ values lower than 1 provide corresponding levels of evidence for the null hypothesis (*H_0_*).

All analyses were conducted using JASP 0.19.3 (JASP Team, 2024).

### 2.2 Results and Discussion

#### 2.2.1 Circumnavigation Side

The frequentist GLMMs revealed a significant effect of Group (χ^2^(1) = 9.197, *p* = 0.002), but no significant effects for Test (χ^2^(1) = 2.452, *p* = 0.117) or Test order (χ^2^(1) = 0.005, *p* = 0.943).

The Bayesian GLMMs corroborated (1) the effect of Group, with the increasing‐group showing a positive effect (β = 0.898, 95% CI [0.295, 1.552]) and the decreasing‐group showing a negative effect. A negative effect indicates a significantly lower probability of choosing a left‐side approach compared to the increasing‐group. Behaviorally, a negative effect represents a reduction or cancellation of the spontaneous left‐side bias following exposure to decreasing numerical sequences. (2) The absence of evidence for significant effects of Test and Test order, with credible intervals including zero. The model's intercept was estimated at β = 0.851 (95% CI [0.157, 1.660]).

#### 2.2.2 Left Preference Index

Chicks trained with the increasing sequence (increasing‐group) showed a significant left‐to‐right approach in both the MNL‐congruent test (M = 76%, 95% CI [0.311, 0.867], SE = 5.973, V = 176.000, *p* = 0.007, rrb = 0.676) and the MNL‐incongruent test (M = 75%, 95% CI [0.191, 0.832], SE = 7.975, V = 168.000, *p* = 0.015, rrb = 0.600), based on one‐sample Wilcoxon test results. The Bayes factor analysis yielded very strong evidence for left‐to‐right searching behavior in the MNL‐congruent test (BF_10_ = 56.869), and moderate evidence in the MNL‐incongruent test (BF_10_ = 8.523). In contrast, the decreasing‐group (8‐5‐2) exhibited no significant spatial preference in either the MNL‐congruent test (M = 43%, 95% CI [−0.720, 0.093], SE = 7.685, V = 64.500, *p* = 0.13) or the MNL‐incongruent test (M = 53%, 95% CI [−0.462, 0.462], SE = 8.852, V = 105.000, *p* = 1.0), with the Bayes factor analysis yielding moderate evidence for the null hypothesis (MNL‐congruent test: BF_10_ = 0.325; MNL‐incongruent test: BF_10_ = 0.248).

**FIGURE 4:**
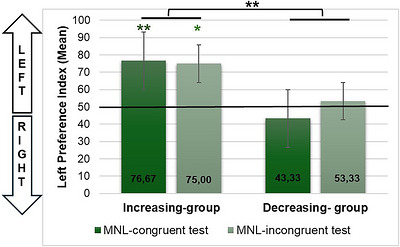
Mean of Left Preference Index for the increasing‐group and the decreasing‐group as a function of Test (MNL‐congruent test, MNL‐incongruent test) in Experiment 1. The horizontal line indicates chance level (50%). Asterisks refer to significant differences from chance level within each group using the Wilcoxon test, and differences between groups using the Mann−Whitney test (*p < 0.05; **p < 0.01).

The one‐tail Mann−Whitney test showed significant between‐group behavior differences in both the MNL‐congruent test (W = 306.000, *p* = 0.001, rrb = −0.530; Bayesian analysis provided very strong evidence: BF_+0_ = 44.885) and the MNL‐incongruent test (W = 264.500, *p* = 0.033, rrb = −0.323; Bayesian analysis provided anecdotal evidence: BF_+0_ = 2.112). Paired‐samples Wilcoxon test found no significant within‐group differences between tests for either the increasing‐group (*p* = 1.0; BF_10_ = 0.238, moderate evidence favoring the null hypothesis) or the decreasing‐group (*p* = 0.16; BF_10_ = 0.523, anecdotal evidence favoring the null hypothesis; Figure 4).

### 2.3 Discussion

To summarize the results of Experiment 1, the significant group effect, the absence of a test effect, and the chance‐level performance of the decreasing‐group supported hypothesis 2. The lack of a test effect allowed us to reject hypothesis 1, while the lack of a significant rightward preference in the decreasing‐group allowed us to reject hypothesis 3.

The absence of a group × test interaction indicated that numerical training did not influence the MNL: Chicks’ performance was independent of congruency of test with the MNL.

These results provided compelling evidence that exposure to increasing or decreasing numerical sequences can influence, but does not fully replace, the spontaneous left‐side bias in the visuospatial exploration of numerosities: Chicks trained with increasing numerical sequences consistently preferred to approach the panels from the left; chicks trained with decreasing numerical sequences reported no spatial preference.

The critical impact of early experiences and learning effects on predispositions aligns with findings in infants, whose spontaneous left‐to‐right bias for increasing numerosity can be enhanced by habituation to horizontal sequences increasing from left to right and cancelled by exposure to right‐to‐left horizontal sequences [10]. However, it is worth highlighting that while infants were habituated with horizontal/lateralized (left−right) numerical sequences, chicks in our study underwent a sagittal (near−far) training with nonlateralized numerical sequences, thus removing spatial confounds from training to test.

## 3 Experiment 2

From Experiment 1, two critical aspects remained unaddressed: (1) the *generalizability* of the previous findings to higher numerical ranges and (2) their *independence from continuous variables* that covary with numerosity. To address these points, in Experiment 2, we used stimuli from a larger numerical range and controlled for total area and density.

### 3.1 Materials and Methods

#### 3.1.1 Subjects and Rearing Conditions

We conducted an a priori power analysis using G*Power (version 3.1.9.4) for a between ANOVA design. The following parameters were established: power  =  0.85; α  =  0.05; f  = 0 .6 (derived from the effect sizes observed in Experiment 1 and converted to f); number of groups = 2 (increasing‐group, decreasing‐group); number of measurements = 2 (MNL‐congruent test, MNL‐incongruent‐test). The results indicated that a minimum of 28 subjects was required. We consider this a conservative estimation with respect to the GLMMs employed in Experiment 2, as GLMMs typically have greater statistical power than traditional ANOVA designs because GLMMs can account for both fixed and random effects.

The design of Experiment 2 comprised 36 male chicks. But due to null responses, six chicks were excluded from the analysis. Thus final sample size was 30 chicks.

The rearing conditions were the same as in Experiment 1.

#### 3.1.2 Apparatus and Stimuli

The experimental setup was nearly the same to that in Experiment 1. The only difference was related to the stimuli, which depicted 8, 20, or 32 red squares. Thus, the largest numerosity used in Experiment 1 was the smallest numerosity in Experiment 2. The specific numerosities were taken from the seminal study on the discovery of spatial−numerical association in day‐old chicks and its relative nature [19].

**FIGURE 5:**
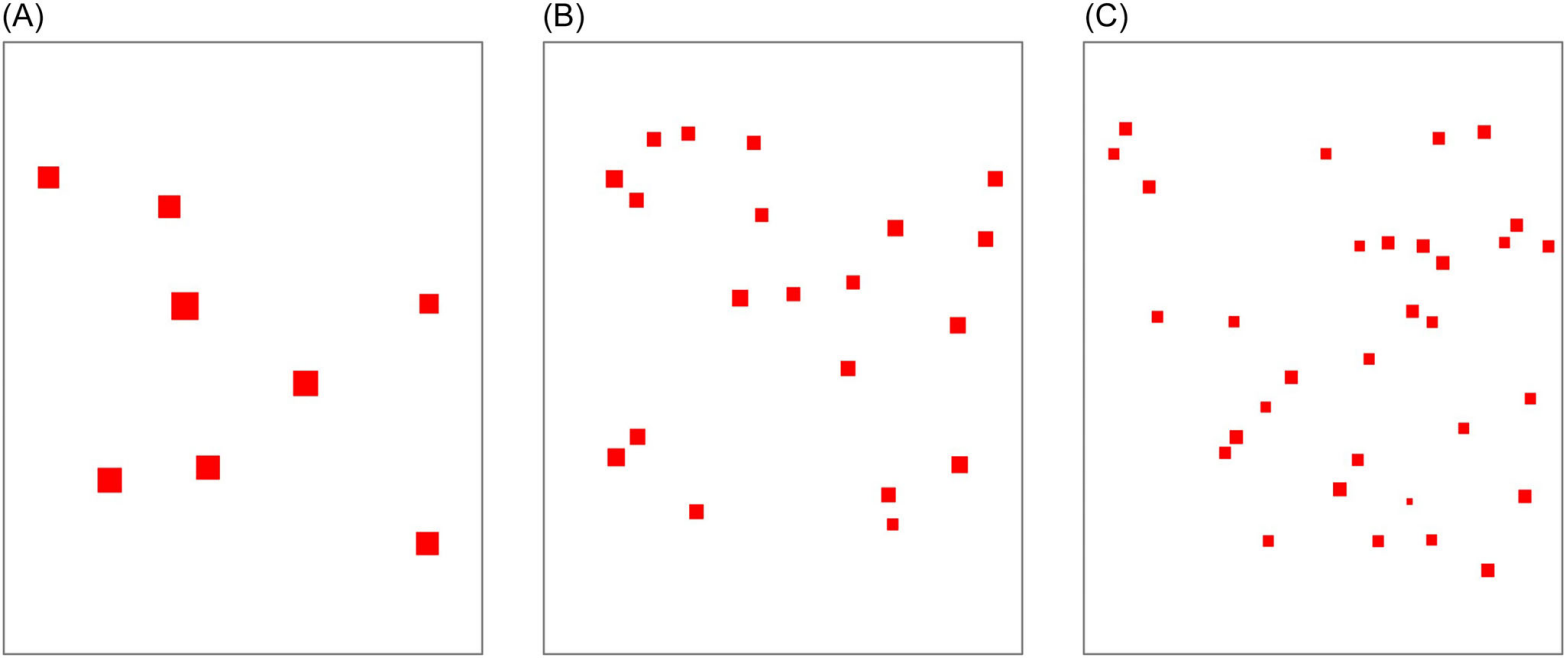
Example of stimuli used in Experiment 2. They displayed 8 (A), 20 (B), or 32 elements (C).

Controlled stimuli were obtained using the Generation of Numerical Elements Images Software (GeNEsIS) [43] with the following parameters: Arena radius (20), radius variability (0.1), shape (square), balance_total area (30 pixels), max distance (1000 pixels), and max radius (100 pixels). The stimuli were then saved by configuring additional settings: Arena shape (square), arena dimension (20 pixels), arena dimension (10 cm), pixel_X screen (1728), pixel_Y screen (2208), width *x* (20 cm), and height *y* (25.5 cm). Total area was identical for all three numerosities (30 pixels). This approach also controlled for density, defined as the proportion of total stimulus area relative to the constant background area (Figure 5). The minimum and maximum distances between the centers of two squares varied for each stimulus: for stimulus 8, 0.7−10 cm; for stimulus 20, 0.4−9.5 cm; and for stimulus 32, 0.2−10.1 cm. For the highest numerosity (32), the individual elements (approx. 0.4 cm) subtended a visual angle of 1.53° at the 15 cm viewing distance. This value is well above the spatial resolution limits (approx. 7.0 cycles per degree) of young chicks [37, 38], ensuring that the subjects could resolve the individual elements even at the highest numerical range.

#### 3.1.3 Procedure

The experimental procedure was identical to that of Experiment 1.

Fifteen chicks underwent pretraining and training with the increasing sequence (8‐20‐32; increasing‐group), and 15 chicks underwent the same phases with the decreasing sequence (32‐20‐8; decreasing‐group). In the increasing‐group, eight chicks completed the MNL‐congruent test (8‐20‐32, from left to right) before the MNL‐incongruent test (32‐20‐8, from left to right), and seven chicks underwent the test in reverse order. In the decreasing‐group, six chicks completed the MNL‐congruent test (8‐20‐32, from left to right) before the MNL‐incongruent test (32‐20‐8, from left to right), and nine chicks underwent the test in reverse order.

#### 3.1.4 Design and Hypotheses

The experimental design was identical to that of Experiment 1.

In Experiment 2, we expected to replicate the findings of Experiment 1, namely, a preference to start circumnavigating the horizontally arranged numerosities from left to right only in the increasing‐group. A replication would document the generalization of the impact of numerical training on the left‐side bias using a different numerical range and controlling for continuous variables.

#### 3.1.5 Analyses

The analysis for Experiment 2 was conducted on 30 chicks (increasing‐group: *n* = 15; decreasing‐group: *n* = 15), with a total of 180 data points (six trials per subject). We performed the same analysis conducted in Experiment 1.

### 3.2 Results and Discussion

#### 3.2.1 Circumnavigation Side

The frequentist GLMMs revealed a significant effect for Group (χ^2^(1) = 4.709, *p* = 0.030), but no significant effects for Test (χ^2^(1) = 0.360, *p* = 0.548) or Test order (χ^2^(1) = 0.118, *p* = 0.731).

The Bayesian GLMMs corroborated (1) the effect of Group, with the increasing‐group showing a positive effect (β = 0.787, 95% CI [0.083, 1.581]) and the decreasing‐group showing a negative effect; and (2) the absence of evidence for significant effects of Test and Test order, with credible intervals including zero. The model's intercept was estimated at β = 1.191 (95% CI [0.447, 2.060]).

#### 3.2.2 Left Preference Index

**FIGURE 6:**
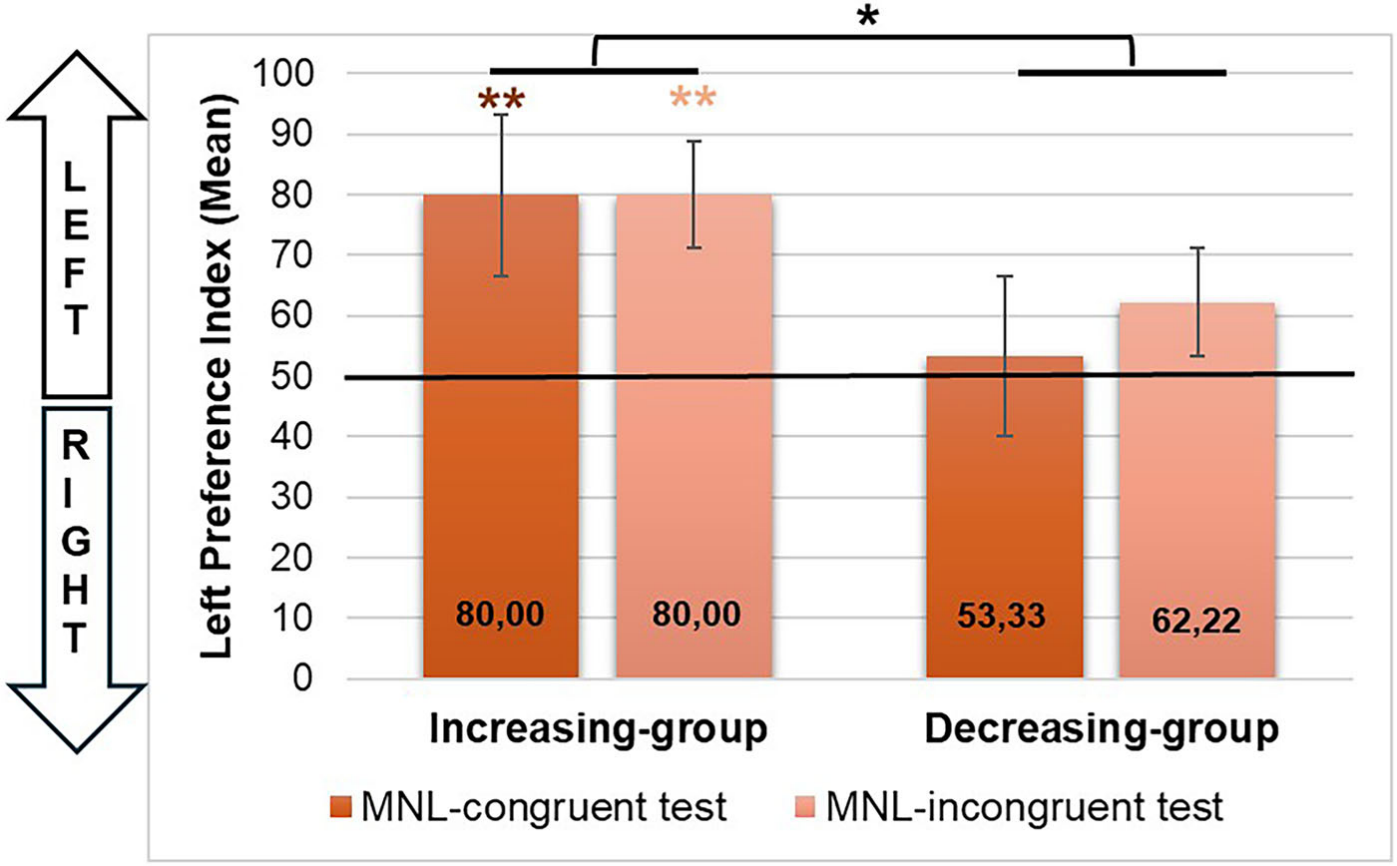
Mean of Left Preference Index for the increasing‐group and decreasing‐group as a function of test (MNL‐congruent test; MNL‐incongruent test) in Experiment 2. The horizontal line indicates chance level (50%). Asterisks refer to significant differences from chance level within each group using the Wilcoxon test, and differences between groups using the Mann−Whitney test (* *p* < 0.05; ** *p* < 0.01).

The increasing‐group showed a significant left‐to‐right approach preference in both tests: MNL‐congruent test (M = 80%, 95% CI [0.632, 0.956], SE = 6.341, V = 112,000, *p* = 0.003, rrb = 0.867) and MNL‐incongruent test (M = 80%, 95% CI [0.428, 0.923], SE = 7.834, V = 106,500, *p* = 0.007, rrb = 0.775), based on one‐sample Wilcoxon test results. This finding was corroborated by Bayes factor analysis, which provided extreme evidence for the MNL‐congruent test (BF_10_ = 103.098) and strong evidence for the MNL‐incongruent test (BF_10_ = 22.978). In contrast, the decreasing‐group exhibited no significant spatial preference in either test: MNL‐congruent test (M = 53%, 95% CI [−0.449, 0.583], SE = 10.690, V = 65,500, *p* = 0.76; BF_10_ = 0.325, moderate evidence favoring the null hypothesis) or MNL‐incongruent test (M = 62%, 95% CI [−0.131, 0.769], SE = 8.524, V = 85,000, *p* = 0.15; BF_10_ = 0.248, moderate evidence favoring the null hypothesis). One‐tail Mann−Whitney test confirmed significant differences between the increasing and decreasing groups in both the MNL‐congruent test (W = 153,500, *p* = 0.038, rrb = 0.364) and the MNL‐incongruent test (W = 151,000, *p* = 0.045, rrb = 0.342). Bayes factor analysis provided moderate evidence for the MNL‐congruent test (BF_+0_ = 3.541) but only anecdotal for the MNL‐incongruent test (BF_+0_ = 1.497). A paired‐samples Wilcoxon test revealed no significant within‐group differences between the two tests for either the increasing‐group (*p* = 0.7) or decreasing‐group (*p* = 0.2; Figure 6). Bayes factor analysis supported no within‐group differences, with moderate and anecdotal evidence for the null hypothesis in the increasing‐group (BF_10_ = 0.238) and decreasing‐group (BF_10_ = 0.523), respectively.

### 3.3 Discussion

To summarize, Experiment 2 replicated and extended the findings from Experiment 1, supporting the conclusion of the influence of numerical training on left‐side bias in day‐old chicks and hypothesis 2. The results demonstrated that the significant/null left‐to‐right bias observed in the increasing‐group/decreasing‐group, respectively, persists even with a larger numerical range (8‐20‐32 instead of 2‐5‐8) and control of continuous variables.

## 4 General Discussion

The present study investigated the impact of exposure to increasing or decreasing numerical sequences on the spontaneous left‐to‐right visual exploration in newborn animals. In two experiments, chicks underwent a training involving either an increasing or a decreasing numerical sequence, sagittally arranged. All chicks were then tested with the same numerosities organized horizontally either in an MNL‐congruent (e.g., 2‐5‐8) or in an MNL‐incongruent (e.g., 8‐5‐2) manner. Experiment 1 revealed a clear preference for circumnavigating both the MNL‐congruent and MNL‐incongruent numerical arrangement from the left in the increasing‐group, and no consistent spatial preference in the decreasing‐group. Experiment 2 replicated this pattern of results using a larger numerical range (8‐20‐32) under control of continuous variables. Notably, the main group effect also emerged when directly comparing the two experiments (see Supplementary Material).

A limitation of the current study is the lack of video recordings for the shaping and training phases, which precludes a retrospective trial‐by‐trial analysis of potential individual side biases during these early stages. However, no systematic side biases were introduced procedurally: The metal stick used to guide the chicks was moved toward the center of the panels, and its use was gradually phased out until the chicks reached the learning criterion independently. Furthermore, previous research utilizing a similar paradigm [20] has demonstrated that the specific side from which a chick circumnavigates a panel is not an informative measure for spatial−numerical associations, as numerical magnitude consistently explains spatial choices, while individual lateral biases do not. The validity of our results is further reinforced by the significant behavioral difference between the two groups: Since the physical apparatus and procedural guidance were identical for all subjects, the observed differences can be attributed solely to the numerical order of the sequences (increasing vs. decreasing) rather than to a learned motor habit or side preference.

Our study indicated that early exposure to increasing or decreasing numerical sequences shapes the spontaneous left‐side bias of day‐old chicks when approaching a series of numerosities. Training with increasing numerical sequences, despite being sagittal, may have activated a spatial mapping in line with the natural left‐to‐right representation of numerical magnitude, thus reinforcing the spontaneous tendency to approach the stimuli from the left. Conversely, sagittal training with decreasing numerical sequences may have induced a right‐to‐left spatial representation, thus weakening the left‐side bias. The findings demonstrate that when numerical training has an incongruent effect with respect to the spontaneous bias in spatial and numerical processing, an interplay between learned and pre‐existing processes may occur already in early development. We found that learning effects on lateralized preferences (1) were induced by experiences with sagittal (i.e., nonlateralized) numerical sequences, and these effects (2) persisted across both MNL‐congruent and MNL‐incongruent tests. The independence of the results from the horizontal numerical arrangement at test suggests that the impact of numerical experience in newborn animals is not a simple stimulus−response association. Rather, it represents a systematic modulation of visuospatial attention. By utilizing sagittal training, we demonstrated that the exposure to numerical sequences can fundamentally alter the spatial biases of the vertebrate brain, making the resulting exploratory behavior robust against immediate contextual changes in stimulus arrangement. Remarkably, even if the training did not require the processing of the numerical aspects of the stimuli, the difference observed between the increasing‐ and decreasing‐groups indicates that chicks processed numerosity. This supports previous evidence that day‐old chicks attend to and memorize the numerical properties of relevant stimuli with which they interact [44, 45].

In addition, the present study replicates hallmark effects with a novel procedure. Specifically, (1) left‐side bias—the percentage of left choices—was significantly above chance; this finding aligns with previous research in humans [1], newborns [2], and animals [3, 4, 30]; and (2) MNL—the impact of increasing and decreasing numerical sequences—is in line with the association of small numerosities with the left and large numerosities with the right [31, 46]. Experiment 2 also corroborated previous findings on the relative nature of spatial−numerical association and its independence from continuous physical variables, such as total area or density [19].

However, other continuous variables, such as total perimeter, convex hull, and interitem distance, were not systematically controlled. To mitigate the influence of these cues, we employed seven different spatial configurations for each numerosity, randomly assigning them to training and test phases to prevent the chicks from relying on fixed visual patterns. Crucially, the significant group effect reported in both experiments provides strong evidence that these physical features were not the primary drivers of behavior: Since both the increasing‐ and decreasing‐groups faced identical physical stimuli at test, the difference in their spatial preferences must be attributed to the numerical sequence experienced during training. A low‐level visual feature that could have played a role is also spatial frequency, defined as the number of dark and light circles per degree of visual angle. The recent brain's asymmetric frequency tuning model proposes the role of hemispheric asymmetry for spatial frequency processing as scaffolding the spatial associations driven by numerosity [47, 48].

Future research should investigate the independence of these induced spatial biases from other continuous variables, their persistence or modifiability with time or new experiences, and their implications for more complex numerical and spatial tasks.

## 5 Conclusions

Experiments 1 and 2 consistently demonstrated that exposure to increasing numerical sequences during training influences the spontaneous left‐side bias in visual exploration of numerical stimuli in newborn animals, regardless of the specific numerical arrangement encountered at test. The consistent approach from the left side by the increasing‐group contrasts with the lack of spatial preference by the decreasing‐group. This effect persisted across different numerical ranges and was independent of total area and density. The observation that spatial preferences can be shaped by early experiences with numerical sequences underscores the fundamental link between spatial and numerical processing, and the plasticity of visuospatial exploration in early development.

In conclusion, our study (1) advances knowledge of the evolutionary origin of the interplay between spatial and numerical processing; (2) represents a pivotal step toward understanding how early experiences associated with numerosity can shape predispositions in animal models; (3) prompts researchers to consider the contextual flexibility of fundamental cognitive processes even in the earliest stages of development; and (4) opens new avenues for investigating the developmental trajectories of spatial and numerical abilities, as well as their potential conservation across species.

## Author Contributions

Conceptualization: A.F., M.M., and R.R.; Methodology: A.F., M.M., and R.R.; Investigation: A.F. and M.M.; Data analysis: A.F., M.M., and L.R.; Writing – original draft: A.F.; Writing – review and editing: A.F., M.M., and R.R.; Supervision: R.R.; Funding acquisition: R.R.

## Conflicts of Interest

The authors declare no conflicts of interest.

## Supporting information




**Supplementary Material**: nyas70325‐sup‐0001‐SupplementaryMaterial.docx

## Data Availability

Data are publicly available at https://osf.io/ae72j/?view_only=e80116deb2d44f149e95d2755eafbe0d
