# Supplementary material for: Numbers Driving Space: Experience With Numerical Sequences Modulates Spatial Behavior in Newborn Chicks (Gallus gallus)
Source: Ann N Y Acad Sci. 2026 Jul 14;1561(1):e70325. doi: 10.1111/nyas.70325 (PMC13366458; doi:10.1111/nyas.70325)
Supplement: Supplementary file 1 — Supplementary Material: nyas70325‐sup‐0001‐SupplementaryMaterial.docx [file NYAS-1561-0-s001.docx]

**Comparing Experiment 1 and 2**

The combined analysis across both experiments utilized a total of 420 data points from 70 subjects.

**Results**

*Circumnavigation Side*. The inclusion of **Experiment** as a fixed factor to the GLMMs corroborated the presence of a significant effect of Group (χ²(1) = 14.382, *p* < .001), but no significant effects for Test (χ²(1) = 3.239, *p* = .072), Test order (χ²(1) = 0.002, *p* = .963), or Experiment (χ²(1) = 0.959, *p* = .327).

The Bayesian GLMMs corroborated **I)** the effect of Group, with the Increasing-group showing a positive effect (β = 0.841, 95% CI [0.398, 1.346]), and the Decreasing-group showing a negative effect; **II)** the absence of evidence for significant effects of Test, Test order, and Experiment, with credible intervals including zero. The model's intercept was estimated at β = 1.018 (95% CI [0.525, 1.579]).

*Left Preference Index*. The Increasing-group showed a significant left-to-right approach preference in both Tests: MNL-congruent tests (M = 78%, 95% CI [0.578, 0.890], SE =4.309, V = 560.000, *p* < 0.001, rrb = 0.778) and MNL-incongruent tests (M = 77%, CI [0.408, 0.832], SE = 5.596, V = 526.500, *p* < 0.001, rrb = 0.671), based on one-sample Wilcoxon test results. Bayes factor analysis provided extreme evidence for directional preference in the Increasing-group (BF₁₀ = 84376.280 in MNL-congruent test; BF₁₀ = 832.028 in MNL-incongruent test). In contrast, the Decreasing-group exhibited no significant spatial preference in either Test sequence: MNL-congruent test (M = 47%, CI [-0.477, 0.236], SE =6.305, V = 271.500, *p* = 0.47) or MNL-incongruent test (M = 57%, CI [-0.216, 0.493], SE =6.198, V = 365.000, *p* = 0.41). These outcomes were sustained by Bayes factor that provided moderate and anecdotal evidence in supporting absence of spatial preference in the Decreasing-group (BF₁₀ = 0.194 in MNL-congruent test; BF₁₀ = 0.334 in MNL-incongruent test).

One-tail Mann-Whitney test confirmed significant between-group differences in both Tests: MNL-congruent test (W = 892.500, *p* < 0.001, rrb = 0.457) and MNL-incongruent test (W = 813.000, *p* = 0.007, rrb = 0.327). Bayes factors provided extreme evidence for MNL-congruent test (BF_+0_ = 289.359) and moderate for the MNL-incongruent test (BF_+0_ = 5.364). Paired-samples Wilcoxon test revealed no significant within-group differences between Test for either the Increasing-group (*p* = 1.0; BF₁₀ = 0.184, moderate evidence favoring the null hypothesis) or Decreasing-group (*p* = 0.07; BF₁₀ = 0.677, anecdotal evidence favoring the null hypothesis; **Figure S1**).


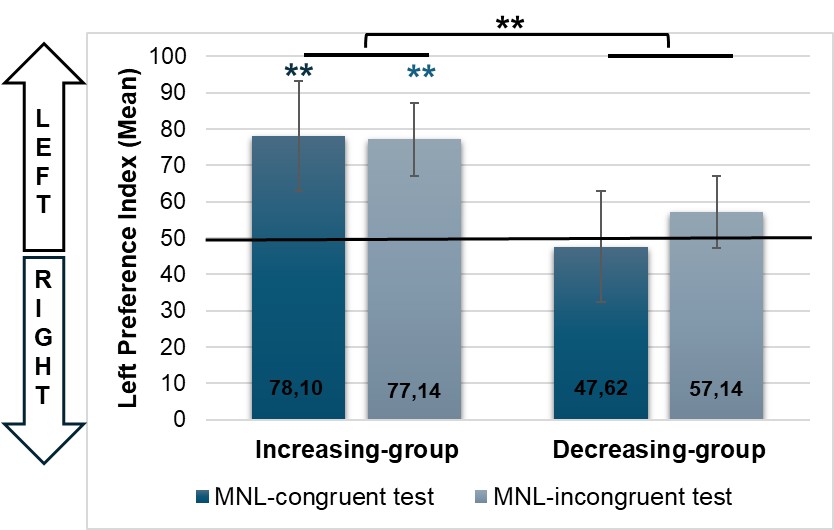


**Figure S1:** Mean of Left Preference Index for the Increasing-group and Decreasing-group as a function of Test (MNL-congruent test, MNL-incongruent test) in all experiments. The horizontal line indicates chance level (50%). Asterisks refer to significant differences from chance level within each group using the Wilcoxon test, and differences between groups using the Mann-Whitney test (*: p < .05; **: p < .01).
